# Image Captioning with Bidirectional Semantic Attention-Based Guiding of Long Short-Term Memory

**DOI:** 10.1007/s11063-018-09973-5

**Published:** 2019-01-11

**Authors:** Pengfei Cao, Zhongyi Yang, Liang Sun, Yanchun Liang, Mary Qu Yang, Renchu Guan

**Affiliations:** 1 Key Laboratory of Symbolic Computation and Knowledge Engineering of Ministry of Education, College of Computer Science and Technology, Jilin University, Changchun 130012, China; 2 College of Computer Science and Technology, Dalian University of Technology, Dalian 116024, China; 3 Zhuhai Laboratory of Key Laboratory of Symbolic Computation and Knowledge Engineering of Ministry of Education, Zhuhai College of Jilin University, Zhuhai 519041, China; 4 MidSouth Bioinformatics Center, University of Arkansas Little Rock, Little Rock 72204, USA; 5 Joint Bioinformatics Ph.D. Program, University of Arkansas Little Rock and University of Arkansas for Medical Sciences, Little Rock 72204, USA; 6 University of Chinese Academy of Sciences, Beijing 100049, China; 7 National Laboratory of Pattern Recognition (NLPR), Institute of Automation Chinese Academy of Sciences, Beijing 100190, China

**Keywords:** Image captioning, Semantic attention mechanism, Convolution neural network, Bidirectional guiding LSTM

## Abstract

Automatically describing contents of an image using natural language has drawn much attention because it not only integrates computer vision and natural language processing but also has practical applications. Using an end-to-end approach, we propose a bidirectional semantic attention-based guiding of long short-term memory (Bag-LSTM) model for image captioning. The proposed model consciously refines image features from previously generated text. By fine-tuning the parameters of convolution neural networks, Bag-LSTM obtains more text-related image features via feedback propagation than other models. As opposed to existing guidance-LSTM methods which directly add image features into each unit of an LSTM block, our fine-tuned model dynamically leverages more text-conditional image features, acquired by the semantic attention mechanism, as guidance information. Moreover, we exploit bidirectional gLSTM as the caption generator, which is capable of learning long term relations between visual features and semantic information by making use of both historical and future contextual information. In addition, variations of the Bag-LSTM model are proposed in an effort to sufficiently describe high-level visual-language interactions. Experiments on the Flickr8k and MSCOCO benchmark datasets demonstrate the effectiveness of the model, as compared with the baseline algorithms, such as it is 51.2% higher than BRNN on CIDEr metric.

## 1 Introduction

Automatically generating a natural language description of an image using a sentence, which is known as image captioning, has emerged as a popular multi-disciplinary task in both academia and industry [7,28]. Pursuing this task not only facilitates theoretical development in machine learning but also has great practical potential; for instance, in performing this task, one can help visually impaired people understand the content of an image. Although it is effortless for a human to describe the content of an image, it is very challenging for machines to do so. This task is difficult because it requires a computer not only to capture the objects and attributes in an image but also to express the semantic relations between them in natural language. To address this problem, deep neural networks with encoder–decoder structures are employed. These deep neural networks mainly consist of two sub-networks: deep convolution neural networks (CNNs) for image representation and recurrent neural networks (RNNs) for language modeling [7,8,10,19,24]. However, a bottleneck facing the encoder–decoder framework is that it is difficult to exploit all the visual information necessary to produce a caption of an image that accurately describes it.

Therefore, Xu et al. [28] introduced an attention mechanism into the encoder–decoder framework to break down this bottleneck by refining the original image features. Although this approach achieved significant improvements in image captioning, several issues remain to be explored. First, some existing baselines employ unidirectional LSTMs as decoders used to generate captions. One of the shortcomings of unidirectional LSTMs is that they only make use of generated textual information, thereby limiting their ability to predict any future context. It is obvious that if a model simultaneously captures both past and future contextual information from a sentence, it will achieve a higher prediction accuracy. Moreover, a semantic attention mechanism [30] has been proposed as an efficient method to address image captioning with attributes. However, the attribute predictor has no learning ability and is separated from the encoder–decoder model. Therefore, it cannot be trained in an end-to-end manner. In addition, visual attention refines visual image features conditioned on previously generated information, thus it has limited ability to exploit both current and future hidden states of RNNs. Some existing methods [7,24] merely input visual representations into RNNs at the first step, or else, the input visual information is time invariant. Such methods have difficulty in exploiting visual information sufficiently enough to generate captioning that is coupled to the image content.

**Fig. 1:**
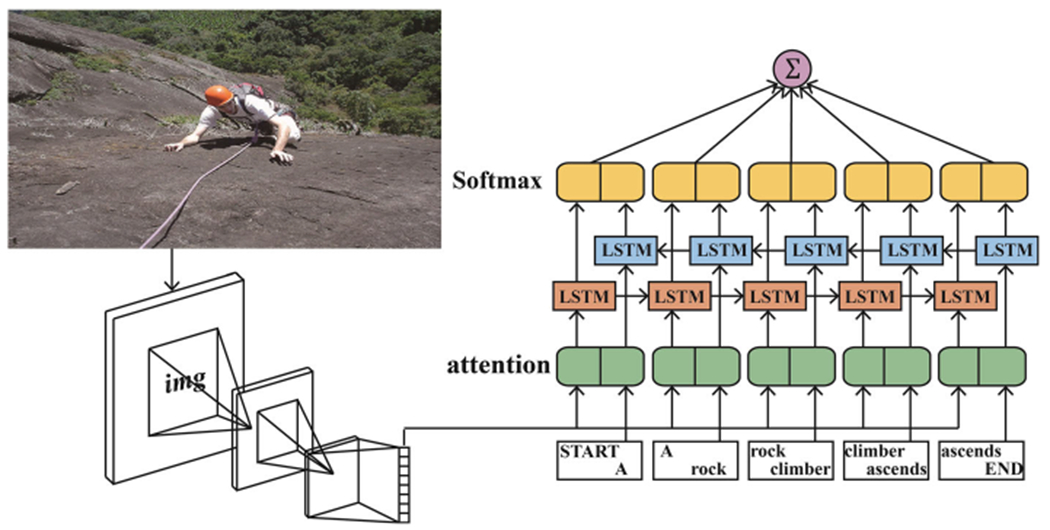
The framework of the proposed model consists of a CNN, a guiding Bi-LSTM, and an attention mechanism

To address the above issues, we propose a novel Bag-LSTM model for effective image captioning. Our model is illustrated in Fig. 1. The main contributions of our paper are as follows:

1. To obtain text-related image features, we propose a new semantic attention mechanism, which can automatically focus on features on demand using generated contextual information. Moreover, the semantic attention mechanism can be trained with the encoder–decoder model in an end-to-end manner.
2. To our knowledge, it is the first work to combine a bidirectional gLSTM (Bi-gLSTM) with an attention mechanism in a model used for image captioning. To learn long term visual-language interactions, the model embeds visual features and contextual information into semantic space by exploiting both historical and future context. The text-conditional image features used as semantic guidance are input into each unit of the Bi-LSTM with the aim of guiding the model towards more relevant predictions of image information.
3. We adjust the structure of the Bag-LSTM to refine the original image features using current hidden state information more than the generated information.
4. The proposed model achieves better performance than all baselines on bench-mark datasets, such as the MSCOCO dataset [17], across several evaluation metrics.

## 2 Related Work

Generally, the existing image captioning approaches can be divided into three categories: sentence-template based methods, retrieval based methods and neural network based methods.

Sentence-template based methods [4,11,16,29] use pre-defined templates to generate sentences by filling detected objects or recognized scenes into templates. Apparently, this method has difficulty in generating flexible captioning and cannot accurately express the relations between objects [6]. Retrieval based methods [12–14] treat image captioning as a retrieval and ranking task. They first find similar images from a large database and then modify the retrieved image sentences to generate a new sentence. The effectiveness of this method is extremely limited when dealing with previously unseen images in datasets [6].

With inspiration from the success of neural networks in machine translation tasks [1], the encoder–decoder framework [7,20,24] has been widely applied in image captioning tasks and has yielded promising results. This framework was first introduced by Kiros et al. [9], who described a multimodal log-bilinear model for image captioning with a fixed context window. Mao et al. [20] proposed a multimodal recurrent neural network for the prediction task where a deep CNN interacted with a deep RNN in a multimodal layer. Later, Vinyals et al. [24] and Donahue et al. [3] used an LSTM network in their model for sentence generation. However, Vinyals et al. [24] only provided visual input at the beginning of processing, while Donahue et al. [3] fed image context information at each step. Still, the input visual information used by Donahue et al. [3] is time invariant. Instead of extracting image features as a single vector from a fully connected layer, Karpathy et al. [7] proposed an alignment model that can generate descriptions of image regions by integrating object detection with a regional-CNN and inferring the alignment between image regions and the outputs of a bidirectional RNN. Jia et al. [5] proposed a gLSTM that added semantic information into each unit of the LSTM block with the aim of guiding the model towards the captioning that is most tightly coupled with the image information presented. Wang et al. [25] employed a bidirectional LSTM as a language model to generate captioning, which enabled them to exploit both long term history and future context in completing the task of automatically generating a caption that describes an image.

Recently, Xu et al. [28] proposed visual attention mechanisms for image captioning, including soft attention and hard attention, in which the model focused on specific regions based on the previous state of an RNN. Although hard attention performed better, its training was very complex. You et al. [30] proposed a semantic attention model with attribute prediction. However, the attribute predictor required separate training. Yang et al. [27] proposed a “reviewer” module to acquire previous attention-related information. A very recent work proposed “Areas of Attention” that modeled the interplay between the state of an RNN, image region descriptors and word embedding vectors by three pairwise interactions [22]. And Liu et al. [18] proposed an image captioning architecture that allows a visual encoder and a language decoder to coherently cooperate in a recurrent manner.

Distinct from these previous models, we propose a Bag-LSTM model that dynamically integrates image features with generated textual information, obtains text-related image features via the semantic attention model and learns long term high-level visual-semantic relations from the model’s history and future context. In addition, the text-related visual features input into each unit of a Bi-LSTM block are expected to guide the LSTMs toward the solution that most closely matches the image content presented to the model.

## 3 Bidirectional Semantic Attention-Based Guidance of Long Short-Term Memory

**Fig. 2:**
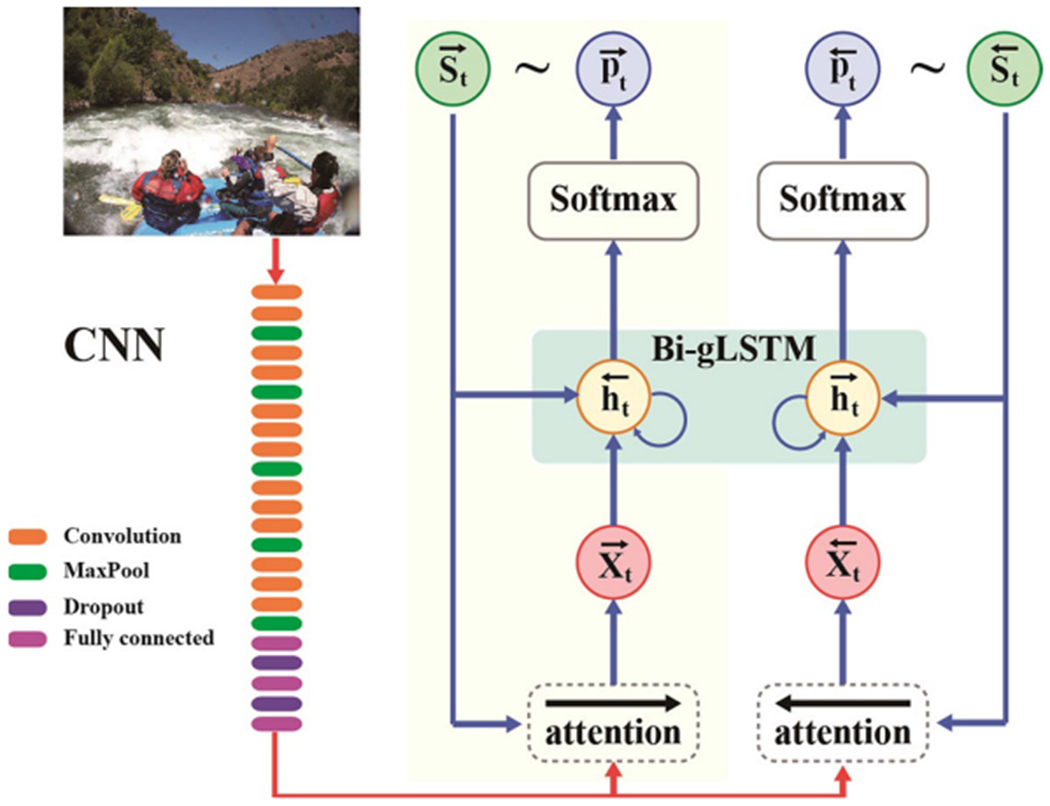
The framework of the Bag-LSTM model: CNN for visual representation, Bi-gLSTM for caption generation and visual features v and generated semantics are combined together using an attention mechanism

In this section, we first introduce the novel model for image captioning holistically, as shown in Fig. 2. Then, we present the structure of our bidirectional semantic attention-based guidance of long short-term memory model (Bag-LSTM) and introduce the intuitions behind it. Finally, we describe the selection algorithm which is used to determine the final sentence presented as a caption for each image.

### 3.1 Overall Framework

This model consists of CNNs, the semantic attention mechanism and the Bi-gLSTM, which processes both left-to-right and right-to-left sequence context. We first replace the unidirectional LSTMs with bidirectional LSTMs, which are capable of learning long term visual-language interactions by exploiting historical and future semantic information. Then, a new and trainable semantic attention model is proposed, which is expected to obtain more text-related image features. Eventually, to make more sufficient use of visual information and to obtain more accurate predictions, we leverage the text-related visual features acquired via semantic attention as guidance information that is input into the LSTMs.

First, we use CNNs to extract image features. Global features acquired from the fully connected layer are expected to provide macroscopic correspondence among multiple objects. Suppose global visual information is denoted by $v\in R^{D}$. A CNN implements its function as:

(1) $$v=W\left[CNN_{\theta }\left(I_{b}\right)\right]+b$$

where **I_b_** denotes the image pixels, *CNN*(**I_b_**) transforms the pixels into *L* dimensional activations and $W\in R^{D\times L}$ and $b\in R^{D}$ are the weight matrix and bias vector, respectively. *CNN* denotes the VggNet-16, which secured the 2nd place in ImageNet Large Scale Visual Recognition Competition 2014 (ILSVRC-2014). ***θ*** represents the parameters.

Then, the image features obtained from the CNN are fed into the semantic attention model to obtain text-related features based on the generated contextual information. Finally, the text-related visual features are injected into the Bi-gLSTM to generate the description of an image.

At time *t*, the Bi-gLSTM hidden state controls the prediction of the *t*-th word **S_t_** from corpus *γ* with a probability $p_{t}\in R^{\left|\gamma \right|}$. The generated word **S_t_** will be fed into the semantic attention model and the Bi-gLSTM in the next step. Unlike previous image captioning methods that only leverage image features at the first step, our model uses the text-related visual features as guidance information that is input into the Bi-LSTM at each step. Specifically, our model is implemented using the following equations:

(2) $$x_{0}=f_{att_{0}}(v)=Wv$$

(3) $$h_{t}=BLSTM\left(1(t>0)⊙h_{t-1},x_{t}\right)$$

(4) $$S_{t}∼p_{t}=Softmax\left(h_{t}\right)$$

(5) $$x_{t}=f_{att}\left(v,\overset{t-1}{\underset{i=0}{\sum }}S_{i}\right),\;t>0$$

(6) $$\text{Sentence}=\phi (\vec{\text{sentence}},\;\overset{\leftarrow }{\text{sentence}})$$

where $W\in R^{D\times D}$ denotes the weight matrix, *f*_*att*_0__ is semantic attention at the first step, $x_{0}\in R^{D}$ denotes the text-related visual features after semantic attention has been applied at the first step, *BLSTM* represents the Bi-gLSTM network, **1**denotes the indicator function, when *t* > 0, the value of the indicator function is 1, otherwise, the value is 0, $\overset{t-1}{\underset{i=0}{\sum }}S_{i}$ is the sum of generated word vectors, $x_{t}\in R^{D}$ are the text-related visual features after semantic attention has been applied at step *t*, $h_{t}\in R^{D}$ is the hidden state of the Bi-gLSTM, $S_{t}\in R^{\left|\gamma \right|}$ represents the generated word at step *t*, *f*_*att*_ is the semantic attention mechanism; *Sentence*, $\vec{\text{sentence}}$ , and $\overset{\leftarrow }{\text{sentence}}$ represent the final caption, as well as the generated captions of the forward and backward LSTMs, respectively; and *Φ* () denotes the selection algorithm.

### 3.2 Semantic Attention Model

Unlike the approaches that directly exploit image features acquired from CNNs described in [7,24], we propose a semantic attention model to generate image features more closely related to textual information and then these features are used as semantic guidance. During the process of generating a caption, image features interact with generated semantics via non-linear layers at each step. Because the visual information becomes more relevant during this process, the generated semantic information also becomes more accurate as processing continues. Therefore, the proposed semantic attention mechanism refines original image features to obtain text-conditional image features by taking the advantages of previously generated textual information.

An additional advantage of the new semantic attention model is that it can be automatically trained with the encoder–decoder model via feedback propagation.

Specifically, given the image features **v** extracted from a fully connected layer of the CNN and the generated word **S_k_**, the proposed semantic attention mechanism in the Bag-LSTM model can be implemented by:

(7) $$x_{t}=Softmax\left(v⊙\left(W\overset{t-1}{\underset{k=0}{\sum }}S_{k}\right)\right)⊙v$$

where **x_t_** denotes the text-related image features at step *t*, $W\in R^{D\times |\gamma |}$ is the embedding matrix for textual features and can be trained by back propagation, and ⊙ denotes element-wise multiplication.

### 3.3 Bidirectional Guiding of Long Short-Term Memory

**Fig. 3:**
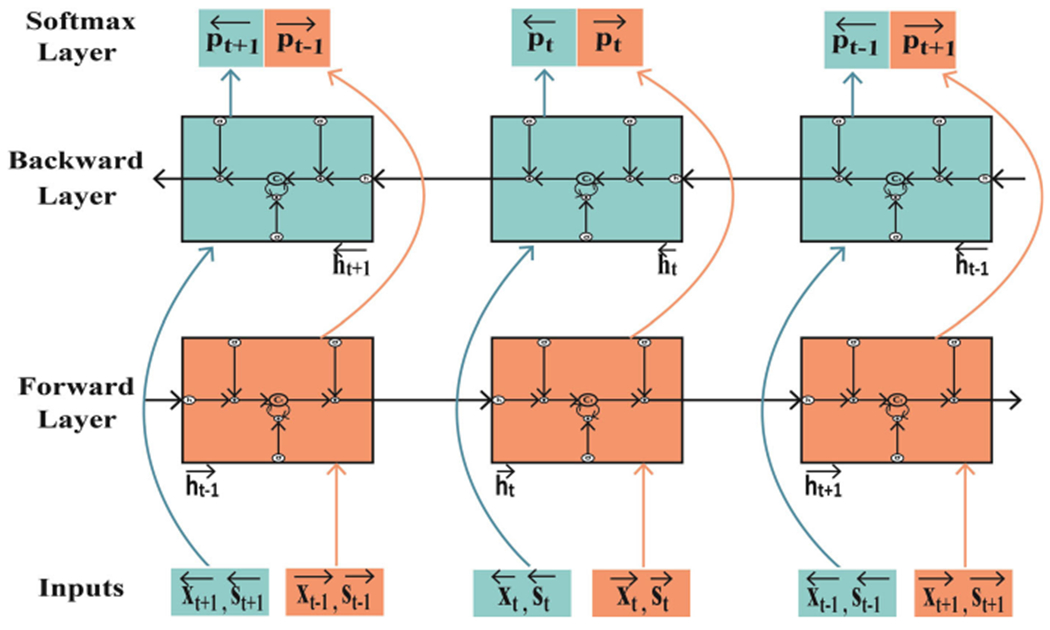
Bidirectional guiding of long short-term memory network (Bi-gLSTM). Each box represents a gLSTM unit. The Bi-LSTMs summarize semantic information from both forward and backward directions

We use gLSTM networks [5] to replace the LSTM networks by feeding the LSTM networks semantic information, which is composed of the text-conditional image features obtained from the semantic attention model. The Bi-gLSTM networks are implemented by introducing a second gLSTM layer, where the hidden-to-hidden connections flow in opposite temporal order based on unidirectional gLSTM networks as shown in Fig. 3.

The Bi-gLSTM network computes the forward and backward hidden sequences denoted as $\vec{h}$ and $\overset{\leftarrow }{h}$, respectively. Taking the forward order as an example, the Bi-gLSTM networks work as follows:

(8) $$\vec{i_{t}}=\sigma \left(W_{ix}\vec{x_{t}}+W_{is}\vec{S_{t}}+W_{ih}\vec{h_{t-1}}+b_{i}\right)$$

(9) $$\vec{f_{t}}=\sigma \left(W_{fx}\vec{x_{t}}+W_{fs}\vec{S_{t}}+W_{fh}\vec{h_{t-1}}+b_{f}\right)$$

(10) $$\vec{o_{t}}=\sigma \left(W_{ox}\vec{x_{t}}+W_{os}\vec{S_{t}}+W_{oh}\vec{h_{t-1}}+b_{o}\right)$$

(11) $$\vec{c_{t}}=\vec{f_{t}}⊙\vec{c_{t-1}}+\vec{i_{t}}⊙h\left(W_{cx}\vec{x_{t}}+W_{cs}\vec{S_{t}}+W_{ch}\vec{h_{t-1}}+b_{c}\right)$$

(12) $$\vec{h_{t}}=\vec{o_{t}}⊙\vec{c_{t}}$$

where **i_t_**, **f_t_** and **o_t_** indicate the input gate, forget gate and output gate, respectively; **c_t_** represents the state of cell memory,they are all *D* dimentional vectors; **x**_**t**_ denotes the text-related image features; **S**_**t**_ stands for the model-generated contextual information; **h**_**t**_ is the hidden state; **W** is the trainable weight matrix; **b** is a bias term; *σ* is a sigmoid activation function, and *h* denotes a hyperbolic tangent function; and ⊙ denotes element-wise multiplication.

To produce the probability distribution of the word from forward and backward prediction, respectively, two softmax layers are employed.

(13) $$\vec{p_{t}}=Softmax\left(\vec{h_{t}};W_{\text{s}},b_{\text{s}}\right),\;\overset{\leftarrow }{p_{t}}=Softmax\left(\overset{\leftarrow }{h_{t}};W_{\text{s}},b_{\text{s}}\right)$$

Then, a selection algorithm is designed to determine the final sentence to be used as the caption for the image. Including the CNN, the semantic attention model and the Bi-gLSTM, the whole model is referred to as Bag-LSTM.

In addition, to extract more image features and learn the visual-language interactions more comprehensively, we adjust the typological structure of the Bag-LSTM network to produce three variants of the model.

#### Bawg-LSTM

**Fig. 4:**
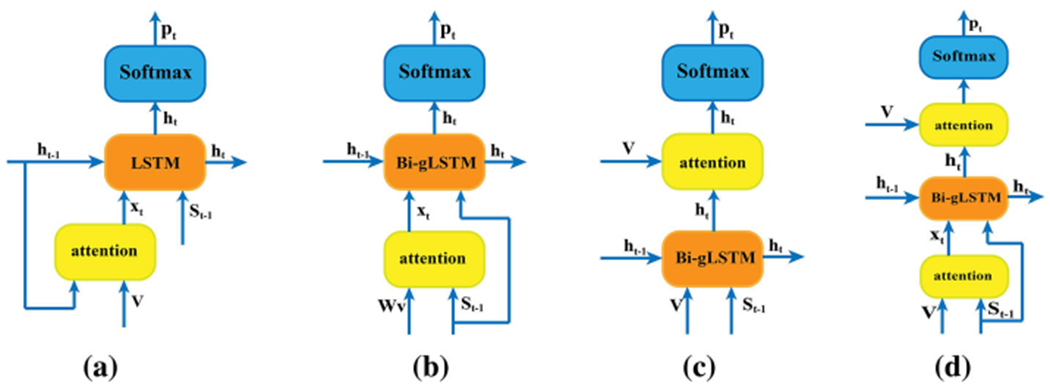
Model comparison **a** attention model proposed in [28], **b** Bawg-LSTM, **c** Bbag-LSTM and **d** Bdag-LSTM are the newly proposed models

Based on Bag-LSTM, Ba**w**g-LSTM is implemented by multiplying the image features by the weight matrix before they are input into the semantic attention module, as shown in Fig. 4b. Clearly, this version of the model enables further refinement of original image features that allows the model to learn more text-related visual representations by feedback propagation to adjust the weight matrix. The semantic attention in Ba**w**g-LSTM is implemented by converting Eq. (7) to:

(14) $$x_{t}=Softmax\left(\left(W_{v}v\right)⊙\left(w_{\text{s}}\overset{t-1}{\underset{k=0}{\sum }}S_{k}\right)\right)⊙v$$

where $W_{v}\in R^{D\times D}$ and $W_{s}\in R^{D\times |\gamma |}$ are weight parameters. Notably, **W**_**s**_ in Eq. (14) is the same as **W** in Eq. (7).

#### Bbag-LSTM

As compared to Bag-LSTM, B**b**ag-LSTM is created by adding the attention mechanism after the Bi-gLSTM layers, as shown in Fig. 4c. After the Bi-gLSTM acquires long term interactions between original visual features and textual information, the hidden state of the Bi-gLSTM is input into semantic attention to obtain a more accurate prediction of the next word of the caption. B**b**ag-LSTM uses the current hidden state of the Bi-gLSTM, **h_t_**, and previously generated information to refine the image features, which is expected to diminish the uncertainty in the next word prediction by complementing the information of the current hidden state. The semantic attention in B**b**ag-LSTM is implemented by the following formula:

(15) $$x_{t}=Softmax\left(v⊙\left(Wh_{t}\right)\right)⊙v$$

where $W\in R^{D\times D}$ is the weight parameter and **h**_**t**_ is the current hidden state of the Bi-gLSTM.

#### Bdag-LSTM

This version of the model synthesizes the advantages of the Bag-LSTM model and the B**b**ag-LSTM model and involves two attention mechanisms, as shown in Fig. 4d. The letter “**d**” in “B**d**ag-LSTM” means “double”. The two attention models are separately located before and after the Bi-gLSTM. The B**d**ag-LSTM uses the first attention model to refine the original visual features and then employs the second attention model to further analyze the image features given the current hidden state. Therefore, this version of the model leverages image and context information more completely and acquires more holistic visual-language relations than other versions. The first attention model is the same as that described by Eq. (7), and the second attention model can be implemented using Eq. (15).

### 3.4 Sentence Selection

The proposed model predicts word *S*_*t*_ with given image *I* and generated words *S*_0:*t*−1_ by *p*(*S_t_*|*S*_0:*t*−1_, *I*) in forward order, or by *p*(*S_t_*|*S*_*t*+1:*T*_, *I*) in backward order. Because the Bag-LSTM model can generate two different caption sentences by processing in two directions, we need to determine the final caption from these two candidates. We devised two methods for selecting a final caption sentence, which are referred to as log based and mean based models. For log based method, we are inspired by the information entropy. The words with small probability have little positive effect on the quality of generated sentences. In addition, the words with large probability (such as the subjects) may simultaneously appear in two different captions. Therefore, there is no need to pay more attention to both of these two kinds of words. Based on the analysis of above, the log based algorithm is proposed to put more emphasis on words of modest probability. For mean based method, we first sum the probability of each word in sentence and then divide the length of the sentence. The mean based algorithm considers each word and is less restricted by the length of sentences. These methods are described by the following equations:

(16) $$H\left(S_{0:T}|I\right)=\max(\frac{\sum_{t=0}^{T_{f}}\left(-\vec{p}\left(\vec{S_{t}}|I\right)\right)\log\left(\vec{p}\left({\vec{S}}_{t}|I\right)\right)}{T_{f}+1},\;\frac{\sum_{t=0}^{T_{b}}\left(-\overset{\leftarrow }{p}\left(\overset{\leftarrow }{S_{t}}|I\right)\right)\log\left(\overset{\leftarrow }{p}\left(\overset{\leftarrow }{S_{t}}|I\right)\right)}{T_{b}+1})$$

(17) $$avgp\left(S_{0:T}|I\right)=\max\left(\frac{\sum_{t=0}^{T_{f}}\vec{p}\left({\vec{S}}_{t}|I\right)}{T_{f}+1},\frac{\sum_{t=0}^{T_{b}}\vec{p}\left({\vec{S}}_{t}|I\right)}{T_{b}+1}\right)$$

where *H*(*S*_0:*T*_|*I*) and *avgp*(*S*_0:*T*_|*I*) indicate the results of the log based and mean based models, respectively; *T_f_* and *T_b_* are the last time step of forward order and backward, respectively, so (*T_f_* + 1) and (*T_b_* + 1) are the length of the sentences generated by forward processing and backward processing, respectively; *I* denotes the image being input to the model; $\vec{p}\left(\vec{S_{t}}|I\right)=\Pi_{j=0}^{t}\vec{p}\left(\vec{S_{j}}|\vec{S_{0}},\vec{S_{1}},\ldots ,\vec{S_{j-1}},I\right)$, and $\overset{\leftarrow }{p}\left(\overset{\leftarrow }{S_{t}}|I\right)=\Pi_{j=t}^{T_{b}}\overset{\leftarrow }{p}\left(\overset{\leftarrow }{S_{j}}|\overset{\leftarrow }{S_{j+1}},\overset{\leftarrow }{S_{j+2}},\ldots ,\overset{\leftarrow }{S_{T_{b}}},I\right)$.

We compute the sentence score of the two different sentences using log score with Eq. (16) and using average score with Eq. (17). Then, the sentence with the higher score is selected as the final result.

## 4 Experiments

### 4.1 Datasets and Evaluation Metrics

We choose the benchmark datasets Flickr8k and MSCOCO to evaluate the performance of our model. Flickr8k and MSCOCO contain 8,000 and 123,000 images, respectively. Each of these images is annotated with 5 sentences. We follow previous work in the way we split these datasets [7]. For the Flickr8k dataset, we used 1000 images for validation and another 1000 images for testing; the rest were used for training. For the MSCOCO dataset, 5000 images were used for validation, another 5000 images for testing and the rest for training. We reported the performance across different evaluation metrics such as BLEU-N (N=1, 2, 3, 4) [21], METEOR [15] and CIDEr [23]. The higher these metrics were, the better were the results.

BLEU (Bilingual Evaluation Understudy) is an algorithm that measures the precision of an n-gram between the generated and reference captions. BLEU-N (N=1, 2, 3, 4) scores can be calculated by the following equation:

(18) $$\log B_{N}=\min\left(1-\frac{r}{c},0\right)+\overset{N}{\underset{n=1}{\sum }}w_{n}\log p_{n}$$

where *r* and *c* are the length of the reference sentence and the generated sentence, respectively; *w*_*n*_ represents the uniform weights, and *p*_*n*_ represents the modified n-gram precisions.

METEOR (Metric for Evaluation of Translation with Explicit Ordering) was also initially used as an evaluation metric in machine translation. In addition to measuring precision, METEOR places emphasis on the recall between the generated and ground truth captions.

CIDEr (Consensus-based Image Description Evaluation) was specifically introduced for evaluating image captioning. It measures the similarity of generated captions to their ground truth sentences. Using human consensus, grammaticality and correctness are also taken into account in this measurement. Therefore, we attach more importance to CIDEr scores in our experiments.

### 4.2 Performance on Flickr8k

We first conducted experiments on Flickr8k data and compared our results with those of five classical algorithms. The results based on different evaluation metrics are shown in Table 1. Flickr8k is a small dataset which introduces difficulties in training complicated models; however, the proposed model still achieves a competitive performance on this dataset. When compared with the bidirectional recurrent neural network (BRNN) model in [7], which is a classical baseline of image captioning model, the Bag-LSTM+mean method achieves better results on all metrics. It is 2.3%, 5.5%, 10.2% and 12.5% higher than BRNN on B-1, B-2, B-3 and B-4, respectively. In addition, when compared with another classical model proposed by Mao et al., it can be seen that the proposed model can significantly improve performance on image captioning tasks. We are aware that the performance of our proposed model is slightly inferior to that of the Google NIC model when using the evaluation metric B-1. We conjecture that GooLeNet is more powerful than VggNet in extracting image features. The fact can be inferred from the performance of these models, as measured by the metrics presented, with these two models using an identical RNN. In addition, our model is more complex than the Google NIC model, so our model suffers more difficulties because it is a deep model being trained with limited data.

It can be seen that the variations of Bag-LSTM, including Bawg-LSTM, Bbag-LSTM and Bdag-LSTM, achieve better performance than the Bag-LSTM model on some concrete metrics. For instance, compared with Bag-LSTM, Bawg-LSTM improves the CIDEr score from 43.6 to 44.2, which reveals that the captioning generated by Bawg-LSTM is more similar to the ground-truth. In addition, we should be aware that our mean based selection algorithm and our log based selection algorithm are both capable of generating superior performance, which demonstrates the effectiveness of the two proposed selection algorithms.

### 4.3 Performance on MSCOCO

To check the performance of our models on a larger dataset, we compare Bag-LSTM and its variations with other models on the MSCOCO dataset. It can be seen from Table 2 that our model outperforms these baselines on all metrics presented. A detailed discussion proceeds as follows:

1. Compared with those models consisting of attention and unidirectional LSTMs (e.g., Soft-Attention and Hard-Attention [28]), our model significantly improves the performance according to all the evaluation metrics presented. For example, the BLEU-4 score is improved from 25.0 to 30.5. Table 2 demonstrates that the scores of Bag-LSTM are 22% higher than those of Hard-Attention and 25.5% higher than those of Soft-Attention. The results indicate that it is more efficient to extract complete visual-semantic relations than to rely on relations that can be uncovered using only unidirectional LSTMs.
2. We note that the proposed model yields significant improvements when compared with those methods that merely exploit Bi-LSTM [25]. Particularly, the Bag-LSTM+mean model increases the BLEU-4 score from 24.4 to 30.5, which is a 25% increase over the performance of the Bi-LSTM model. Therefore, our model reveals that having visual information and semantic context interact via an attention mechanism can make contributions toward producing better captions.
3. In addition, we note that CIDEr scores are improved significantly in our models, which suggests that the caption sentences predicted by our models are very similar to the target captions for the images presented. For example, the Bag-LSTM+mean model and the Bawg-LSTM+mean model have CIDEr scores 4.1% higher than those of the Sentence-condition model proposed in [31]. The CIDEr scores of these models are also 51.2% higher than those of the BRNN model in [7]. These results demonstrate that our Bag-LSTM model is more effective than the other models at image captioning tasks.
4. By comparing variations of the Bag-LSTM model, we realize that the Bag-LSTM and B**b**ag-LSTM models achieve better results than the B**d**ag-LSTM model. For instance, the results achieved by Bag-LSTM are 7% higher than the results generated by B**d**ag-LSTM according to the BLEU-4 metric. In addition, B**b**ag-LSTM achieves the best BLEU-1 result. We conjecture that the more complicated structure of the B**d**ag-LSTM model contributes to its relatively poorer performance.

### 4.4 Analysis of Sentence Selection Algorithms

As described in Sect. 3.4, we propose two selection algorithms to compute sentence scores for determining the final caption. To quantitatively eveluate the quality of the two proposed algorithms and traditional method, we conduct a comparison experiment. Table 3 lists the comparative results of the two proposed sentence selection algorithms and the traditional method with the same model on MSCOCO dataset. The traditional method named as sum based model listed in the table selects a final caption by merely according to $\max\left(\overset{T_{f}}{\underset{t=0}{\sum }}\vec{p}\left(\vec{S_{t}}|I\right),\overset{T_{b}}{\underset{t=0}{\sum }}\overset{\leftarrow }{p}\left(\overset{\leftarrow }{S_{t}}|I\right)\right)$. If the lengths of the generated sentences are quite different, the sentence with more words generally has higher score by only computing $\overset{T}{\underset{t=0}{\sum }}p\left(S_{t}|I\right)$. Therefore, the traditional method will have difficulty in making right decisions. The two selection algorithms proposed for our model can alleviate this problem caused by the different length of generated sentences via considering the length and select a more accurate caption than the traditional method. From the results, we can observe that the proposed selection algorithms achieve the best performance than the traditional method on almost every metric, which clearly demenstrates the effectiveness of the proposed selection algorithms.

### 4.5 Visualization

**Fig. 5:**
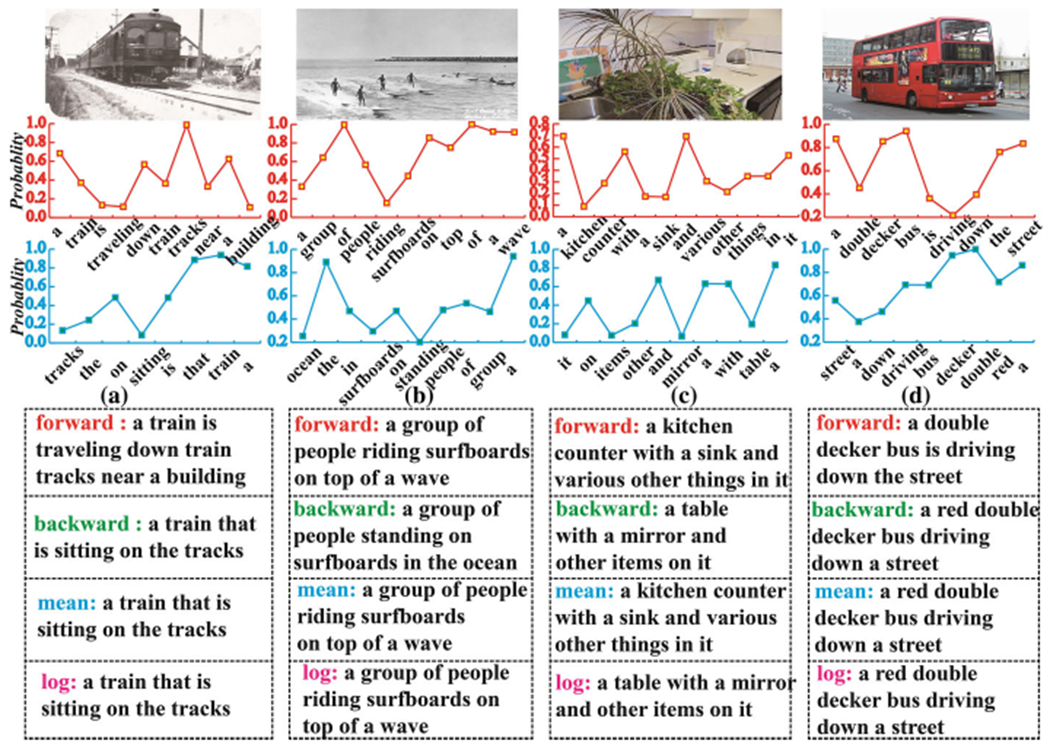
Examples from the MSCOCO dataset, which visualize the generation of captions

In Fig. 5, we selected four representative captioning examples to help visualize the generation of natural language description of image content with our model. The second and third rows show the predicted word probabilities of the Bag-LSTM using forward and backward order processing, respectively. The sixth and seventh rows represent the final caption chosen by the two different selection algorithms: the log based and mean based models.

Because the Bag-LSTM model generates sentences in both forward and backward directions, the length of captions generated by these two processing streams may be different; examples of such cases are shown in Fig. 5a, b. We also found that the semantics captured by the forward sentence may be different from those captured by the backward sentence. For example, in Fig. 5c, the forward sentence describes “kitchen counter” and “sink” while the backward sentence captures “table” and “mirror”. This occurs when a predicted word is the same for both processing streams, but the corresponding probabilities for this word in the separate streams are different. For example, in Fig. 5d, the probability of “bus” is approximately 0.9 in the forward directional processing stream; however, the computations result in a probability 0.7 for “bus” in the backward directional processing stream. This discrepancy occurs because the attention mechanism produces different word probabilities based on different textual information generated in the two separate processing streams.

In addition, these two different selection algorithms can generate different sentences, allowing our model to select a more appropriate sentence than those selected by models using unidirectional LSTMs. For example, in Fig. 5, our model enables the generation of a caption sentence that is very similar to the ground-truth. This example illustrates the proposed model’s enhanced ability to synthesize visual and linguistic information.

**Fig. 6:**
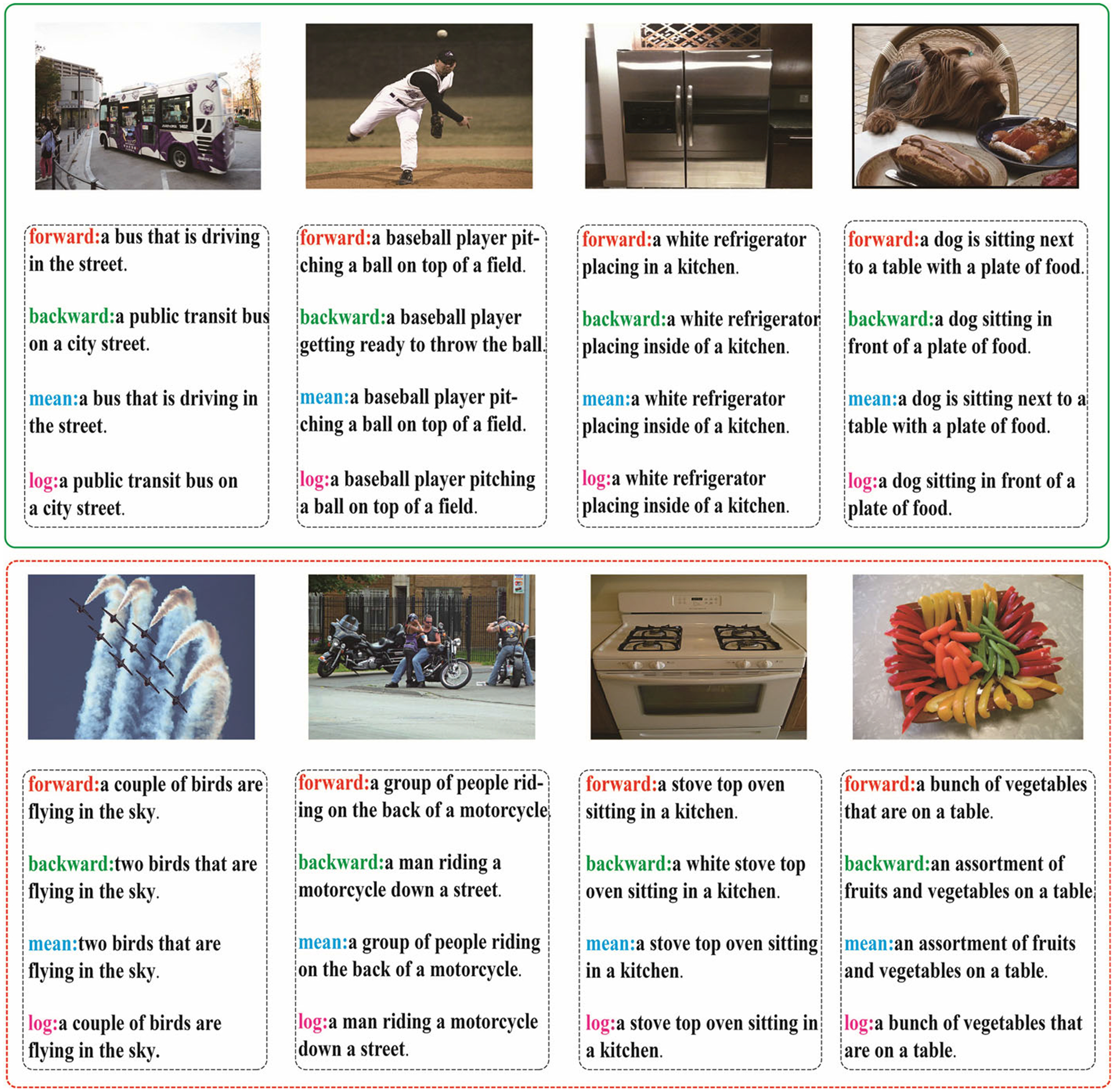
Qualitative results for images with high values of loss function on MSCOCO testing split. The top four examples(green solid box) shows that the proposed model can generate acceptable captions. The bottom four examples(red dashed box) indicate the model can be misled by incorrect visual information. (Color figure online)

We have shown some positive examples about the caption result in Fig. 5. We also sample some testing images with high values of loss function from MSCOCO, as shown in Fig. 6. We can find that the proposed model still enables to generate acceptable results, as shown by the examples in the green box. However, incorrect visual information can disrupt the model, particularly key visual features. For example, for the left-most example in the red dashed box, the model generates “bird” as key visual feature rather than “airplanes”. We think this is due to the lack of relevant examples in training split, and we believe the errors could be solved by introducing more instructive training data in the future.

### 4.6 Efficiency and Discussion

We implemented our model on Torch framework. In our experiment, we use the Adam optimizer with learning rate of 0.4 × 10^−3^ for language model and 0.1 × 10^−4^ for the CNN. We trained our model in two stages. In the first stage, we fix the weights of CNN to pre-train on ImageNet. In the second stage, we back-propagate the loss from Bi-gLSTM to the CNN. All of our experiments were conducted on Ubuntu 14.04, 24G RAM, and single GTX Titan X with 12G memory. On the largest dataset (MSCOCO), our Bag-LSTM took within 3 days, which is as efficient as the classical soft attention model proposed by Bengio et al. [28].

Because the proposed model is relatively deep and complex, it is very possible to be overfitting. In oder to prevent overfitting problem, we adopted several effective techniques such as fine-tuning the parameters of CNN and dropout method. Additionally, we observed the value of loss function on the training set and validation set. If the value of loss function decreased on the training set, however, increasing on the validation set, we will stop the training procedure.

## 5 Conclusion and Future Work

In this work, we proposed a novel model for image captioning, referred to as Bag-LSTM, which performed better than all baselines on benchmark datasets across different evaluation metrics. Bag-LSTM combines a semantic attention mechanism and bidirectional gLSTMs. It not only achieves interactions between visual image features and semantic information but also takes both historical and future contexts into account when generating captions. Next, we designed several variations of Bag-LSTM to sufficiently leverage visual and sentential information in generating image captions. In addition, we qualitatively visualized the procedure of our proposed model for generating a caption sentence at consecutive steps. In the future, we plan to apply our model to other datasets and tasks, such as video captioning and visual question answering.

## Figures and Tables

**Table 1:** Performance of the proposed model on Flickr8k across BLEU-N (N=1, 2, 3, 4), METEOR and CIDEr

Dataset	Model	B-1	B-2	B-3	B-4	METEOR	CIDEr
Flickr8k	BRNN [7]	57.9	38.3	24.5	16.0	–	–
	Mao et al. [20]	58.0	28.0	23.0	–	–	–
	Google NIC [24]	**63.0**	41.0	27.0	–	–	–
	VggNet+RNN [22]	56.2	37.5	24.5	16.6	–	–
	GooLeNet+RNN [22]	56.5	38.5	27.7	16.3	–	–
	Bag-LSTM+mean	59.2	40.4	27.0	**18.0**	**18.3**	**43.6**
	Bag-LSTM+log	58.7	40.7	26.3	17.6	18.1	42.8
	Bawg-LSTM+mean	**59.7**	40.5	**28.1**	17.5	18.0	**44.2**
	Bawg-LSTM+log	58.5	**41.4**	27.4	17.7	17.8	43.6
	Bbag-LSTM+mean	59.3	**41.2**	**27.9**	17.0	17.6	42.9
	Bbag-LSTM+log	59.5	40.7	26.9	**18.2**	**18.6**	42.4
	Bdag-LSTM+mean	58.3	39.7	26.7	17.8	18.0	42.1
	Bdag-LSTM+log	58.1	40.4	26.4	17.5	17.7	42.3

(–) indicates unreported scores. The numbers in bold are the top 2 results of each metric

**Table 2:** Performance of the proposed model on MSCOCO compared with other baselines across multiple evaluation metrics

Dataset	Model	B-1	B-2	B-3	B-4	METEOR	CIDEr
MSCOCO	Google NIC [24]	66.6	46.1	32.9	24.6	–	–
	BRNN [7]	62.5	45.0	32.1	23.0	19.5	66.0
	Log Bilinear [9]	70.8	48.9	34.4	24.3	20.0	–
	Bi-LSTM [25]	67.2	49.2	35.2	24.4	–	–
	ATT-FCN [30]	70.9	53.7	40.2	30.4	24.3	–
	LRCN [3]	62.8	46.1	32.9	24.6	–	–
	Soft-Attention [28]	70.7	49.2	34.4	24.3	23.9	–
	Hard-Attention [28]	71.8	50.4	35.7	25.0	23.0	–
	Sentence-condition [31]	**72.0**	54.6	40.4	29.8	24.5	95.9
	Pedersoli et al. [22]	71.0	30.1	–	–	24.5	93.7
	RIC with STL [18]	68.7	47.8	33.1	22.0	20.5	–
	G-MLE [2]	–	–	39.3	29.9	24.8	**102.0**
	G-GAN [2]	–	–	30.5	20.7	22.4	79.5
	CNN+CNN [26]	68.5	51.1	36.9	26.7	23.4	84.4
	Bag-LSTM+mean	71.7	54.5	**40.8**	**30.5**	**25.3**	**99.8**
	Bag-LSTM+log	71.9	54.5	40.0	29.1	24.3	96.2
	Bawg-LSTM+mean	71.9	54.5	**40.5**	**30.2**	**25.3**	**99.8**
	Bawg-LSTM+log	**72.0**	**54.9**	40.2	29.2	24.6	97.9
	Bbag-LSTM+mean	71.1	53.8	40.1	30.0	24.7	97.7
	Bbag-LSTM+log	**72.3**	**54.8**	40.2	29.3	24.4	97.5
	Bdag-LSTM+mean	70.6	53.7	39.7	29.8	24.9	97.7
	Bdag-LSTM+log	71.6	53.7	39.2	28.5	24.3	96.2

The numbers in bold are the top 2 results of each metric

**Table 3:** Performance of the proposed sentence selection algorithms compared with the traditional selection method on MSCOCO dataset

Dataset	Model	B-1	B-2	B-3	B-4	METEOR	CIDEr
MSCOCO	Bag-LSTM+mean	71.7	54.5	**40.8**	**30.5**	**25.3**	**99.8**
	Bag-LSTM+log	**71.9**	54.5	40.0	29.1	24.3	96.2
	Bag-LSTM+sum	71.7	**54.8**	40.3	29.6	24.5	97.7
	Bawg-LSTM+mean	71.9	54.5	**40.5**	**30.2**	**25.3**	**99.8**
	Bawg-LSTM+log	**72.0**	**54.9**	40.2	29.2	24.6	97.9
	Bawg-LSTM+sum	71.9	54.6	40.5	29.6	24.4	97.8
	Bbag-LSTM+mean	71.1	53.8	40.1	**30.0**	**24.7**	**97.7**
	Bbag-LSTM+log	**72.3**	**54.8**	40.2	29.3	24.4	97.5
	Bbag-LSTM+sum	71.4	53.9	**40.3**	29.5	24.3	95.4
	Bdag-LSTM+mean	70.6	**53.7**	39.7	**29.8**	**24.9**	**97.7**
	Bdag-LSTM+log	**71.6**	53.7	39.2	28.5	24.3	96.2
	Bdag-LSTM+sum	71.5	53.6	**40.0**	29.4	24.1	94.9

The best results of each model on each metric are marked in bold
